# The G20 emission projections to 2030 improved since the Paris Agreement, but only slightly

**DOI:** 10.1007/s11027-022-10018-5

**Published:** 2022-07-14

**Authors:** Leonardo Nascimento, Takeshi Kuramochi, Niklas Höhne

**Affiliations:** 1 NewClimate Institute, Cologne, Germany; 2 Environmental Systems Analysis Group, Wageningen University & Research, Wageningen, the Netherlands; 3 Copernicus Institute of Sustainable Development, Utrecht University, Utrecht, the Netherlands

**Keywords:** Climate change mitigation, Greenhouse gas emissions, Adopted policy scenario, Climate policy, Paris Agreement, Stocktake

## Abstract

Many years passed since the adoption of the Paris Agreement, which invites countries to determine their own contributions to climate change mitigation efforts. The Agreement does not offer a standard to measure progress but relies on a process of periodic stocktakes to inform ambition-raising cycles. To contribute to this process, we compare 2021 greenhouse gas emission projections up to 2030 against equivalent projections prepared back in 2015. Both sets of projections were prepared using the same bottom-up modelling approach that accounts for adopted policies at the time. We find that 2021 projections for the G20 as a group are almost 15% lower (approximately 6 GtCO_2_eq) in 2030 than projected in 2015. Annual emissions grow 1% slower in the coming decade than projected in 2015. This slower growth mostly stems from the adoption of new policies and updated expectations on technology uptake and economic growth. However, around one-quarter of these changes are explained by the effects of the COVID-19 pandemic on short-term emissions and economic forecasts. These factors combined result in substantially lower emission projections for India, the European Union plus the UK (EU27 + UK), the Unites States, Russia, Saudi Arabia, and South Africa. We observe a remarkable change in South African projections that changed from a substantial increase to now a decline, driven in part by the planned phase-out of most of its coal-based power. Emissions in India are projected to grow slower than in 2015 and in Indonesia faster, but emissions per capita in both countries remain below 5 tCO_2_eq in 2030, while those in the EU27 + UK decline faster than expected in 2015 and probably cross the 5 tCO_2_eq threshold before 2030. Projected emissions per capita in Australia, Canada, Saudi Arabia, and the United States are now lower than projected in 2015 but remain above 15 tCO_2_eq in 2030. Although emission projections for the G20 improved since 2015, collectively they still slightly increase until 2030 and remain insufficient to meet the Paris Agreement temperature goals. The G20 must urgently and drastically improve adopted policies and actions to limit the end-of-century warming to 1.5 °C.

**Supplementary Information:**

The online version contains supplementary material available at 10.1007/s11027-022-10018-5.

## Introduction

More than two decades have passed since major emitting countries had a mandate to limit their greenhouse-gas (GHG) emissions (Kyoto Protocol to the United Nations Framework Convention on Climate Change (UNFCCC), 1997). Since then, global GHG emissions have almost doubled and the effects of climate change intensified (IPCC 2021; Olivier & Peters 2021; Tubiello 2020). The long-term temperature goals also became stricter, from 2 °C at the time of the 2009 Copenhagen Accord to ‘well below 2 °C’ and ‘efforts to limit the temperature increase to 1.5 °C’ in the 2015 Paris Agreement. The efforts necessary to reach these goals increase substantially the longer countries wait to curb their emissions (Höhne et al. 2020).

Under the Paris Agreement, countries are invited to submit Nationally Determined Contributions (NDCs), which are pledges that reflect countries’ own interpretation of a fair contribution to the challenge of reducing global emissions and keeping end-of-century warming below 1.5 °C (UNFCCC 2015). There is no common-agreed standard to measure the adequacy of NDCs or the actual progress towards them. Instead, the UNFCCC process relies on periodic stocktakes that shall ‘inform Parties in updating and enhancing, in a nationally determined manner, their actions’ (UNFCCC 2018). To evaluate progress over time is essential to make the Paris Agreement ambition-raising mechanism work.

Since the Paris Agreement was adopted, several countries updated their NDCs, which vary in content and implied absolute emission levels but collectively result in emissions lower than the original ones (Climate Action Tracker 2021b; den Elzen et al. 2021a; UNFCCC 2021). Countries now also announce long-term pledges to reach net zero emissions (Fankhauser et al. 2022; Rogelj et al. 2021). Temperature estimates based on meeting NDCs and net zero targets show an increase in the likelihood of limiting end of century warming temperature increase to 1.5 °C (Höhne et al. 2021; Keramidas et al. 2021; Meinshausen et al. 2022). Countries’ pledges are not equivalent to actions and still fail to secure the global temperature goals but got a boost since the adoption of the Paris Agreement.

Several developments since 2015 affect countries’ ability to meet these pledges. The adoption of each additional climate policy likely reduces national emission intensity (Eskander & Fankhauser 2020). However, the addition of policies alone does not ensure their collective effectiveness (Dubash 2020). Policies remain absent in important mitigation areas (Nascimento et al. 2021) and a mismatch between policy adoption and implementation is observed in key emitters (Silva Junior et al. 2021). Non-policy factors influence emissions as well. The global COVID-19 pandemic, for example, resulted in short-term emission decrease and a global economic downturn (Le Quéré et al. 2020; Z. Liu et al. 2020). Yet, despite the multiple calls to use this moment to increase low-carbon investments, current recovery spending remains insufficient to put countries in a low-carbon trajectory (e.g. Andrijevic et al. 2020; Barbier 2020; Hans et al. 2022; Rochedo et al. 2021). To periodically track changes in emission projections under adopted policies, not only pledges, is fundamental to assess progress towards meeting the goals of the Paris Agreement.

Projected global emissions under adopted policies lead to higher emissions when compared to pledges (den Elzen et al. 2021b). Diverse integrated assessment models show that recent policy-based emission projections remain insufficient to meet global temperature goals (Sognnaes et al. 2021). The median of emissions across studies that use distinct quantification methodologies indicate that global emissions under adopted policies have not yet peaked and are not expected to do so before 2030 (den Elzen et al. 2021b). Policies are sometimes also insufficient to meet countries’ own NDCs (den Elzen et al. 2019; Kuramochi et al. 2021). Even though these results vary across countries, research suggests that collectively countries must implement substantial additional policies and actions to keep global temperature targets within reach.

Although research analysing the collective effect of adopted policies exists, the progress of national emission projections is unclear. More precisely, the change in projections under adopted policies for individual countries, between 2015 and 2021, has not been quantified to date. Höhne et al. (2020) indicate whether 2030 emissions are lower or higher when comparing 2015 to 2020 projections but focus exclusively on trends in a few major emitters. Here, we aim to fill this research gap by investigating how emission projections, which include most recently adopted policies and the COVID-19 pandemic, progressed in the G20 countries^1^ since the adoption of the Paris Agreement. We compare emission projections prepared in 2015 to projections prepared in 2021; both sets include the effect of the policies adopted at that time. We measure whether absolute emission levels in 2030 changed since 2015, evaluate how countries’ estimates contribute to these changes, and discuss factors that influence observed changes.

Our research is mostly descriptive as it does not attempt to attribute changes in emissions to specific national or international developments. For example, it does not quantify the effect of individual policies or changes in macro-economic outlooks. Instead, it presents national emission trajectories up to 2030 and discusses factors explaining changes between projections developed in 2015 and 2021.

## Data and methods

### Definition of policy scenario and data sources

All emission projections presented in our analysis can be termed ‘adopted policies scenarios’ or ‘current policy scenario’. They are based on the full implementation of adopted climate and energy policies but exclude policies that were only planned or considered when projections were prepared. The 2015 projections include policies adopted at the latest by 2015, and the 2021 projections have 2021 as cut-off date. NDC and other policy targets are not included unless they are supported by adopted policies or that sufficient evidence of their implementation exists. Due to legislative and data available differences across countries, the policy-selection criteria differ for each country. Our definition is compatible with other studies (den Elzen et al. 2019; Kuramochi et al. 2021). Here, we avoid the commonly used term ‘current policy scenario’ and use ‘adopted policy scenario’ instead because we discuss projections developed at different points in time. Policies in force in 2015 were ‘adopted’ at the time but are not necessarily ‘current’, which implies that they are still in force.

For this paper, we compiled emission projections from the Climate Action Tracker (CAT) project to which the authors contributed since its inception (Climate Action Tracker 2015, 2021a). The CAT provides yearly updates to its ‘current policy scenario’ for all countries analysed here. CAT tracks country’s climate change mitigation efforts since 2011 and is a well-established source of emission trajectories (Höhne et al. 2011). Its data has also been used in a number of scientific publications (e.g. Ou et al. 2021; Rogelj et al. 2016) and is one of the major inputs to the UNEP emission gap report (UNEP 2021).

The CAT quantification method departs from an external reference emission scenario and is complemented with add-on calculations that estimate the effect of policies on emission projections. The inclusion of individual policies depends on the availability of quantifiable impact indicators and likelihood of implementation. We provided a range of emissions for several countries, but not all. This is mostly a result of still limited data availability in some cases, especially with regards to policy implementation and macro-economic drivers, such as the economic growth assumptions that underpins energy demand. Our projections are then harmonised to the latest available historical data, which varies across countries due to distinct reporting requirements (Tab S1). A more detailed description of the quantification method is available elsewhere (Fekete et al. 2021).

Projections developed in 2021 include the effect of the COVID-19 pandemic. We assumed that emission intensity over GDP remains the same as it would under adopted policies excluding the impact of COVID-19 and that emission reductions are induced by a slowdown in GDP growth. Whenever available, we reviewed and included external estimates for the effect of the pandemic on 2020 emissions (Friedlingstein et al. 2021; Z. Liu et al. 2020). In this research, we focused on emission projections *including* the effect of the COVID-19 pandemic. However, the counterfactual scenario *excluding* the effect of COVID-19 is available and used to estimate the magnitude of the pandemic effect on the changes in projections between 2015 and 2021.

Here, we reported all emissions in carbon dioxide-equivalents (CO_2_e) using 100-year global warming potentials (GWPs) from the IPCC Fourth Assessment Report (AR4) (IPCC 2007). All CAT projections developed in 2021 are expressed in AR4 GWP terms using a country-specific approach, which includes a gas-by-gas conversion whenever possible. Older projections, that use GWPs from earlier IPCC Assessment Reports, were converted to AR4 GWPs using a fixed conversion factor based on the emission ratio in the latest historical year. The conversion factor was extracted from a common database but calculated per country (Gütschow et al. 2021).

We focused on the progress in reducing emissions in energy, industry, agriculture, and waste sectors since 2015 and excluded land use, land-use change, and forestry (LULULCF) emissions from our analysis. Including LULUCF emissions is important for research that explores whether countries are on track to meet their self-determined targets or to reach net zero emissions (Fyson & Jeffery 2019; Grassi et al. 2017). However, from a decarbonisation perspective, emissions in all sectors must be substantially reduced. Increasing LULUCF emission sinks, in parallel to reducing emissions in the other sectors, is important but outside the scope of this analysis. Additionally, emissions from LULUCF are notably uncertain, and accounting methods vary greatly (Dooley & Gupta 2017; Krug 2018). This increases the uncertainty on emission projections. However, we note that addressing land-use-related emissions is fundamental to keep the goal of Paris Agreement within reach (Fyson & Jeffery 2019; van Soest et al. 2021).

National population data is based on the United Nations World Population Prospects, medium fertility scenario (UN 2019).

### Analysis of emission progressions

We assessed the progression of 2030 emission projections for each G20 member by calculating differences between the latest projections (2021) and those developed in the year of the Paris Agreement (2015). We analysed several indicators to analyse this progression. First, we calculated the difference in 2030 emissions to estimate the absolute reduction in projected emissions. This metric clarifies whether the G20 group 2030 emissions are higher or lower and which countries’ estimates explain most of the change. We also analysed full emission trajectories for all countries in terms of absolute and per capita emissions to identify changes in trends and emission-peak years. We calculated the percentage change in emissions in comparison to the year of adoption of the Paris Agreement to improve comparability across countries and progress compared to a base year. Finally, we also calculated the yearly percentage change rates for both sets of projections (2015 and 2021) averaged between 2021 and 2030. This metric focuses on the rate instead the absolute change in the coming decade. We removed both upper and lower fifth percentile of the yearly change rate distribution to avoid including abrupt and significant variations, e.g. those induced by lockdown measures.

We analysed key developments that took place in the G20 since 2015, such as the adoption of new policies and the COVID-19 pandemic, and affected emission projections. Diverse initiatives have also led to the availability of more robust and up-to-date historical emission information. Improvements in official inventory reporting, especially by non-Annex-I countries, and the availability of third-party datasets allow for recent trends to be included in our emission estimates (Friedlingstein et al. 2021; Gütschow et al. 2016; UNFCCC 2016). Decrease of key mitigation options costs have also affected external scenarios used in the modelling, even if with a delay (Xiao et al. 2021). Several policies have been adopted since the first projections (Nascimento et al. 2021). We broadly discussed how these developments affect projections and presented a non-exhaustive list of reasons for the changes observed in each G20 country.

**Fig. 1:**
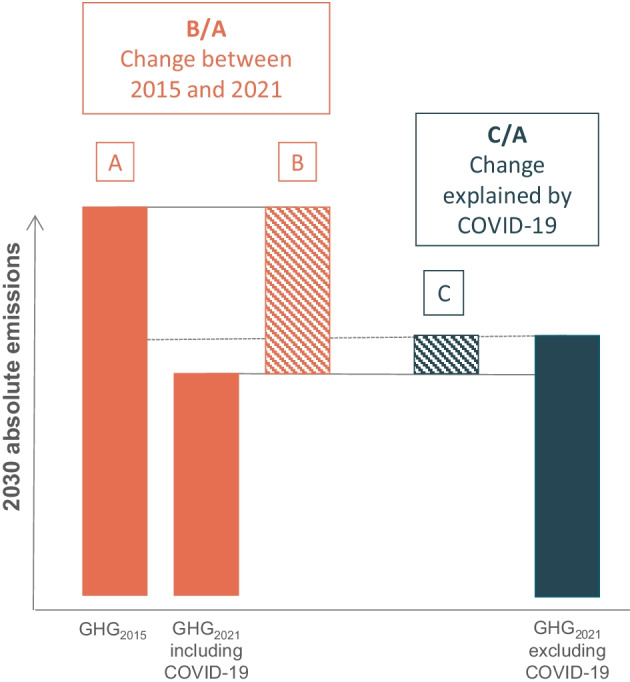
Approach to calculate change in 2030 absolute emissions per country between projections developed in 2015 (GHG_2015_) and 2021 (GHG_2021_) and estimate the contribution of COVID-19 to the change observed

We also compared emission projections *excluding* the effect of the COVID-19 pandemic to emission projections *including* the effect of the pandemic, both developed in 2021 (Fig. 1). By comparing our 2021 projections to the counterfactual, excluding COVID-19 scenario, we singled out the pandemic’s contribution to the changes observed. We calculated the difference between projections including and excluding COVID-19 per country and compared it to the value of 2030 emissions developed in 2015.

## Results

### Progression of emission projections up to 2030

**Fig. 2:**
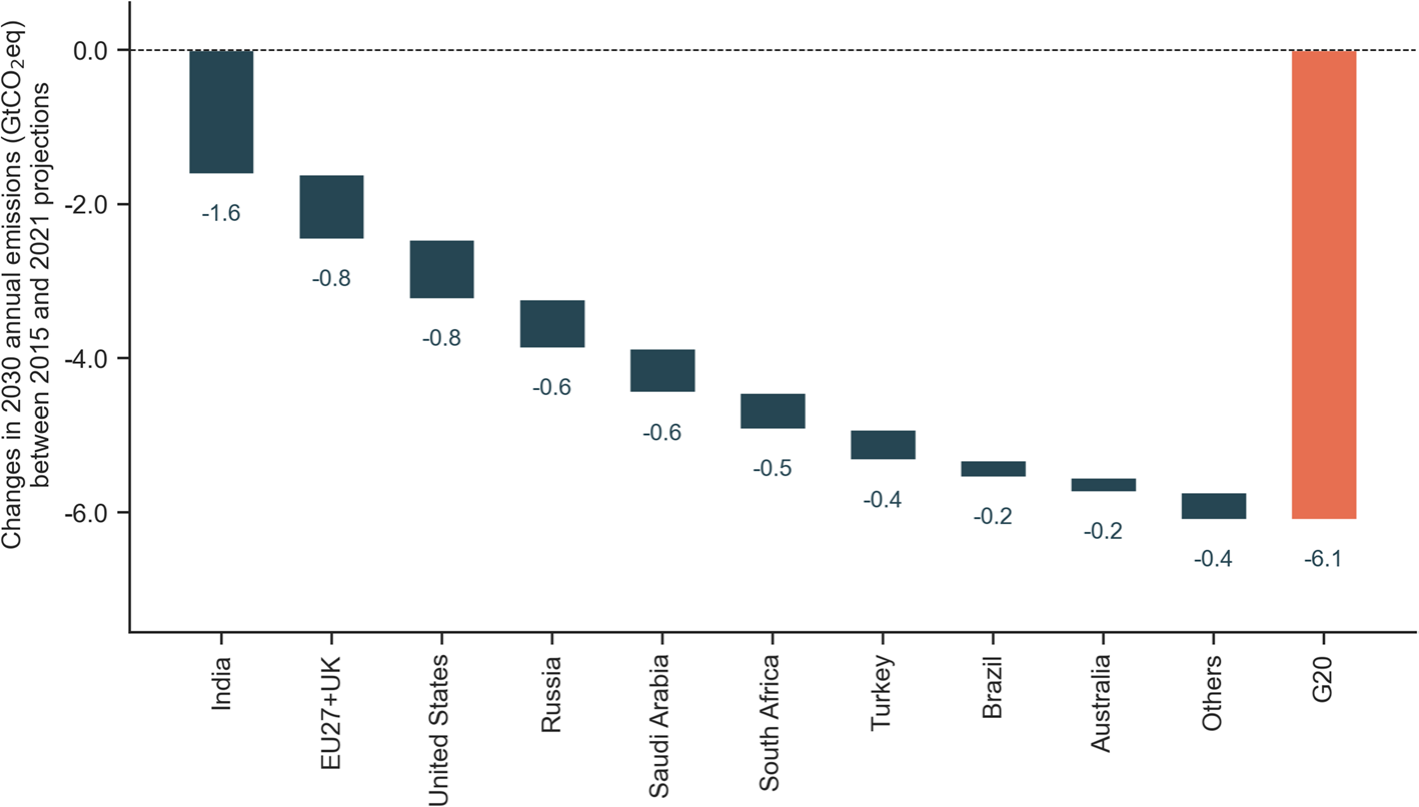
Change in 2030 absolute emission projections. Negative values indicate that projections developed in 2021 are lower than those from 2015. Values based on the middle of the projection range

Absolute 2030 emissions from the 2021 projections are expected to be 6.1 GtCO_2_eq (range: 5.9–6.3 GtCO_2_eq) or 15% lower than those projected in 2015 (Fig. 2). The countries whose projections are more than 0.5 GtCO_2_eq lower in 2030 are India, the EU27 + UK, the Unites States, Russia, Saudi Arabia, and South Africa. GHG emission inventory revisions have varying effects across countries but do not substantially affect this finding. For example, inventory revisions result in lower historical, pre-2010 emissions in Mexico and Russia but higher emissions in Saudi Arabia and Japan. Aggregated changes in inventories were at least two orders of magnitude lower than the reduction observed in 2030 projections. The aggregated difference in historical emissions between the two projections sets (2015 and 2021) is approximately 200 and 10 MtCO_2_e in 2010 and 2015, respectively. The COVID-19 pandemic has resulted in substantial changes, which are discussed in more detail below (Section 3.6).

**Fig. 3:**
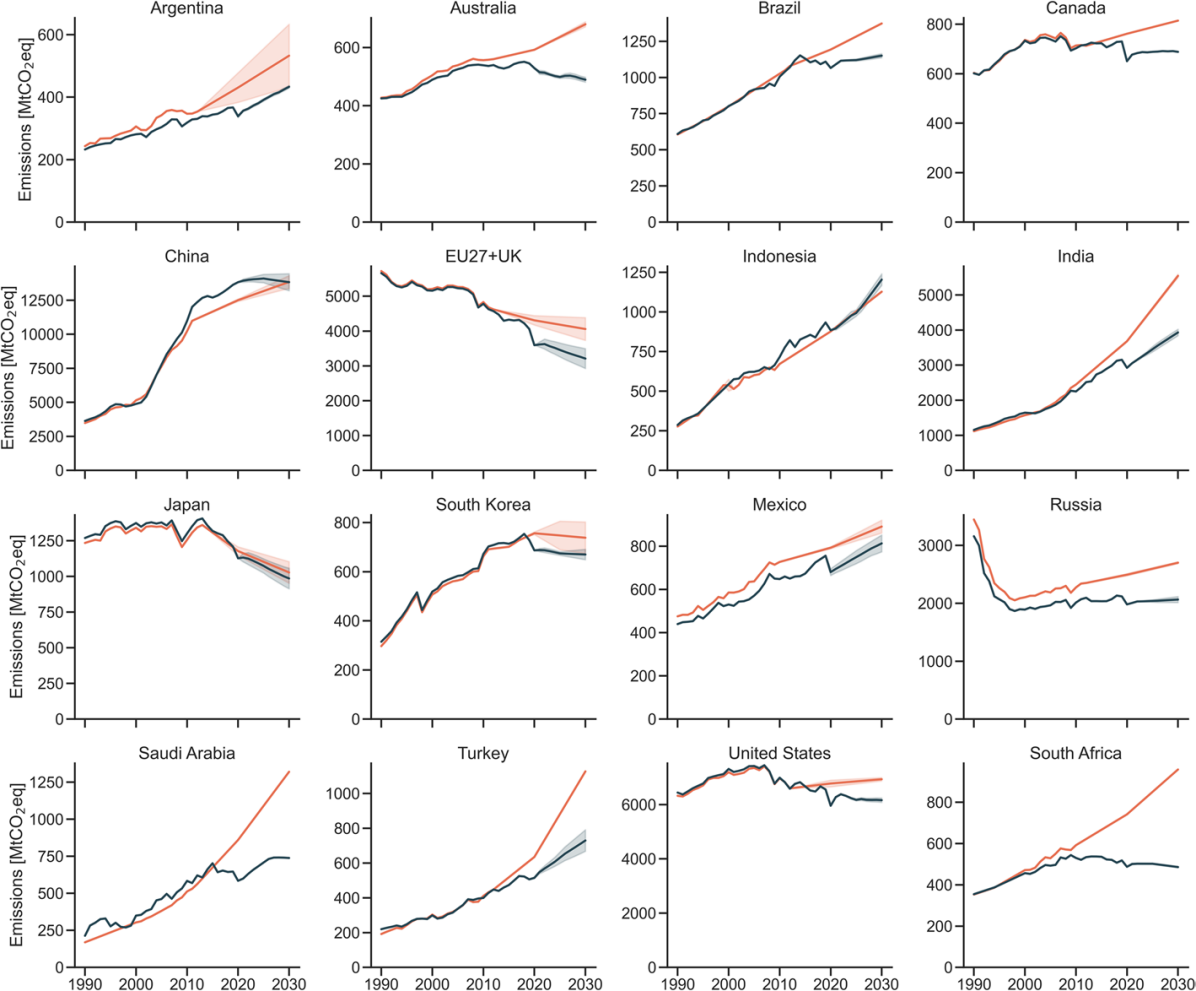
Emission trajectories developed in 2015 (orange) and 2021 (blue) for the G20. Mind the y-axis for each country graph. Emissions exclude LULUCF and are reported in AR4 GWP-100

We do not observe major shifts in G20 countries’ emission projections between 2015 and 2021, with few noteworthy exceptions (Fig. 3). Although the level varies, we observe a shift from increase to decrease in absolute emissions in South Africa, Australia, Canada, and the United States. We do not observe such shifts in other countries. Emission projections developed in 2015 already showed decreasing emissions post-2020 for the EU27 + UK but show a faster decrease rate now. Emissions in Japan remain on a similar downward trajectory. We observe that other countries increase their emissions but at a slower pace now (Argentina, Brazil, India, Mexico, Russia, Saudi Arabia, and Turkey). South Korea’s 2021 and 2015 projections show a similar decreasing trend for post-2020 emissions, but the absolute emission level is lower in 2030. In China, historical emissions have increased substantially, but emissions are no longer expected to consistently increase up to 2030. Indonesia is the only G20 country where the emission estimate for 2030 is now higher than before.

### Per capita emissions

**Fig. 4:**
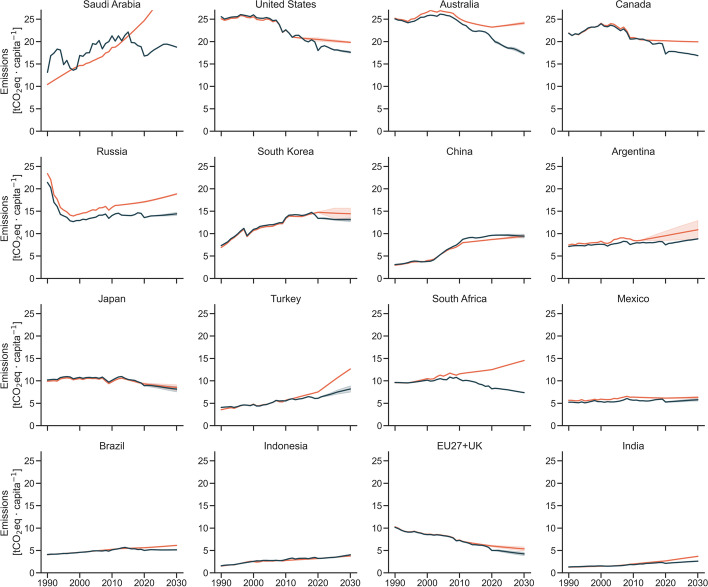
Emissions per capita trajectories developed in 2015 (orange) and 2021 (blue) for the G20 countries. Countries are sorted by emissions per capita levels in 2030. Emissions exclude LULUCF and are reported in AR4 GWP-100

Per capita emission trajectories reveal astonishing differences across the G20 (Fig. 4). Emissions per capita in India and Indonesia are expected to remain below 5 tCO_2_eq per capita in 2030. In India, even with low per capita emissions, emission projections in 2021 indicate a slowdown in growth in comparison to 2015 projections. But in Indonesia, emissions are expected to grow faster than projected back in 2015. The EU27 + UK is the only emitter with decreasing emissions expected to be below 5 tCO_2_eq per capita in 2030. Emissions in most G20 countries are expected to remain between 5 and 15 tCO_2_eq per capita in 2030. South Africa and Japan have now similar emissions per capita levels. In South Africa, emissions per capita are now on a declining trajectory. In Japan, emission projections were already in a declining trend back in 2015, and this has not substantially changed since. Emissions per capita in several other countries have stalled (Argentina, China, Mexico, and Russia) or at least slowed down (Turkey). Emissions per capita are decreasing but remain above 15 tCO_2_eq per capita in 2030 in Canada, Australia, the United States, and Saudi Arabia. In the G20, emissions per capita tend to grow at a slower pace than absolute emissions (Fig S1) due to an overall growing population. In most G20 countries, no growth in absolute emissions implies decline in per capita emissions.

### Expected peaking year

 Since global emissions need to peak immediately to be in line with global scenarios that limit temperature increase to 1.5 °C, we analyse whether individual countries’ emissions have peaked and if so when. The EU27 + UK is the only country analysed where emissions have peaked before 1990. It is followed by the United States, which peaked its emissions between 2000 and 2010. However, emission decrease in the United States is expected to slow down in the coming decade. Trajectories of Australia, Japan, and South Africa indicate that emissions have peaked between 2010 and 2020. To monitor actual emissions in the coming years is necessary to confirm whether these latter three countries have indeed already peaked their emissions and whether they will maintain a sustained decrease. Together, these five countries are responsible for one-third of the G20 emissions.

In the remaining eleven countries, emissions have not peaked. China’s projections indicates that it will peak in the coming decade. In Brazil, Russia Mexico, and Argentina, we expect a moderate increase in emissions. However, no policies in force indicate that emissions will peak before 2030. In South Korea and Canada, emissions are on a slightly decreasing trend. These seven countries are responsible for approximately half of the G20 emissions. In India, Indonesia, Saudi Arabia, and Turkey, emissions are projected to remain on a strong upwards trend.

### Comparison to 2015 base year

**Fig. 5:**
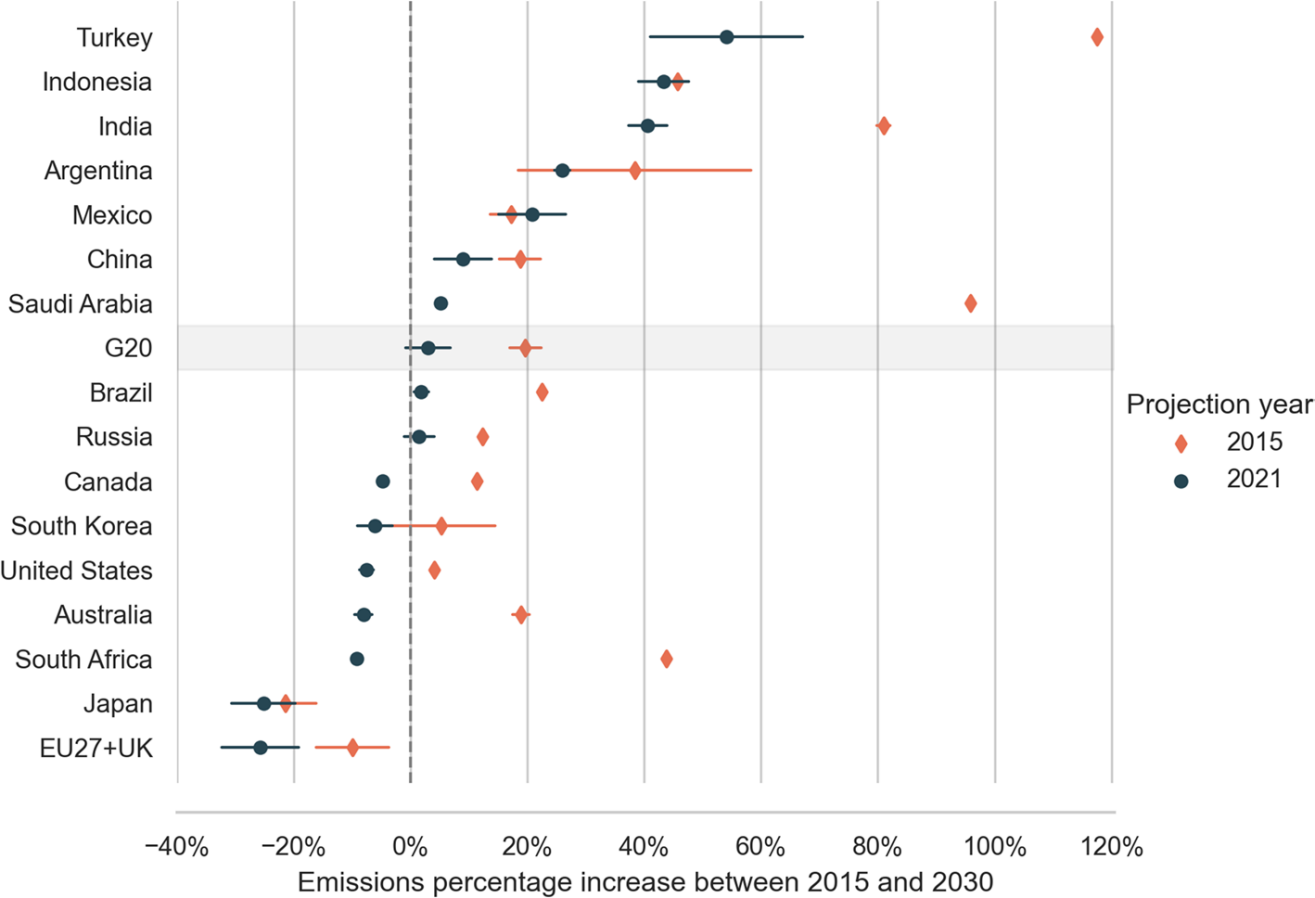
Emissions in 2030 compared to 2015 levels. The bars indicate the range of emissions for each set of projections. Positive numbers indicate an increase compared to 2015

Emission projections developed in 2015 indicate that only two G20 countries, the EU27 + UK and Japan, were unambiguously on track to reduce their emissions in comparison to that year (Fig. 5). Both countries had their total GHG emissions below 2015 levels in the older set of projections. In 2021, the number of countries with emissions below 2015 increased and now covers almost half of the G20 countries. Australia, Canada, the EU27 + UK, Japan, South Korea, South Africa, and the United States are expected to reduce their emissions below 2015 levels by 2030. Japan’s reductions are quite substantial because 2015 emission values represent a peak that resulted from fossil-based replacing nuclear-based electricity after the Fukushima accident in 2011. We expect India, Indonesia, and Turkey to increase their emissions by almost half between 2015 and 2030. Emissions in the G20 are now expected to remain between 1% below and 7% above 2015 levels by 2030. This is down from a 17–22% increase calculated based on 2015 projections.

### Growth rates between 2021 and 2030

**Fig. 6:**
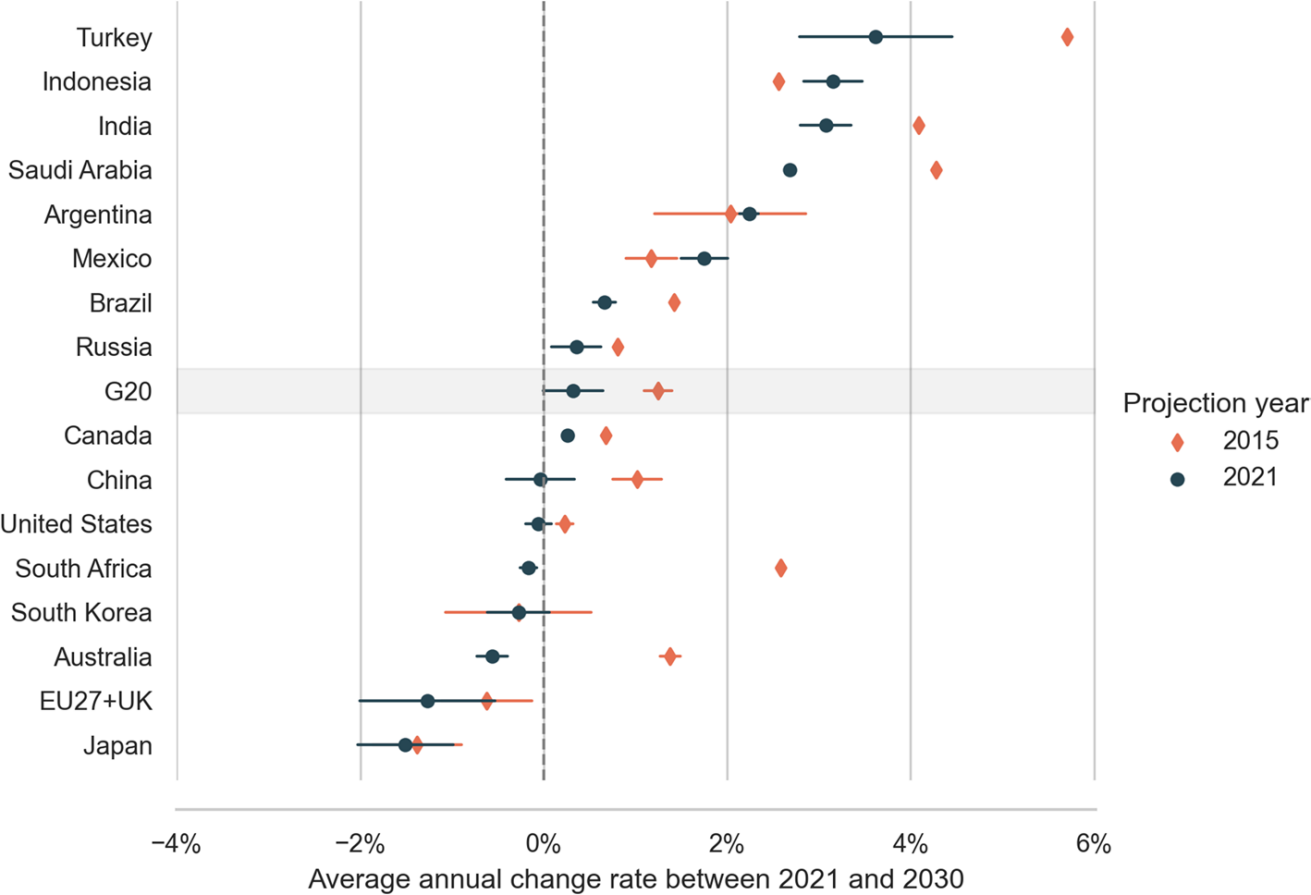
Average yearly change rate in GHG emission projections between 2021 and 2030. Emissions are expected to grow faster in most recent projections for Indonesia, Mexico, and Argentina

The G20 emissions are expected to grow slower between 2021 and 2030 than expected in 2015 (Fig. 6). The average annual growth rate is reduced from 1.2 (range: 1.1–1.4%) in 2015 projections to 0.3% (range: 0.0–0.6%) in 2021 projections. Today, more countries are expected to decrease their emissions. We observe negative change rates in the coming decade for Japan, the EU27 + UK, Australia, South Korea, and South Africa. Two additional countries decrease their emissions when we consider the full range of emissions: China and the United States. However, most countries (nine) are still expected to increase their emissions.

We do not observe a substantial change in emission trajectories as required to meet the Paris Agreement temperature goals. In both sets of projections (2015 and 2021), the distribution of emissions change rates in the 2020s is not significantly different from the change rates in the 2010s (Fig S2). This means that we do not expect currently adopted policies to substantially alter emissions trajectories in the 2020s when compared to the previous decade.

### Factors influencing emission projections

Here, we present a non-exhaustive list of factors that can explain changes in emission projections for each G20 country (Table 1).

**Table 1:** Non-exhaustive list of reasons for change in 2030 emissions between projections prepared in 2015 and 2021. Percentage reduction figures are rounded to the nearest 5%

Country	2030 change (B/A in Fig. 1)	2030 change COVID-19 (C/A in Fig. 1)	Reasons for emission projection change
Argentina	− 15%	− 5%	Lower 2030 emissions result in part from updated historical data and the effect of COVID-19. However, the average emission growth rate in the coming decade is slightly higher. This is attributed to the slower-than-expected uptake of renewable energy, caused in part by limited effectiveness of currently adopted policies intended to foster renewable electricity uptake (Ruggeri & Garrido 2021)
Australia	− 30%	− 5%	Australia’s emission projections are now declining, in comparison to an increase expected back in 2015. Improvements in climate action are mostly driven by subnational actors (Christoff & Eckersley 2021). Lower emissions are hardly a result of improved national policy. Australia has consistently supported fossil fuels and rolled back important climate policies (Crowley 2021)
Brazil	− 15%	0%	Emissions are projected to grow slower than projected in 2015. This is mostly attributed to updates in economic forecasts, especially after the 2015 recession, and adoption of additional policies, such as Brazil’s latest biofuel support program (Denny 2020; IMF 2014, 2021; Sicsú et al. 2021). These findings do exclude LULUCF emissions. Recent LULUCF trends indicate an increase in deforestation-induced emissions and would counteract some of this reduction (SEEG 2021; Silva Junior et al. 2021)
Canada	− 15%	0%	The most recent estimates show a significant drop in 2020 emissions. This is induced by the introduction of new regulations to reduce emissions from oil and gas exploration and production (Government of Canada 2018). The lower emission growth projected in the coming decade is attributed to additional energy and climate policies (Government of Canada 2021)
China	0%	0%	The 2010 historical emissions used in 2015 (CDIAC 2012; IEA 2014; US EPA 2012) were 7% lower than those used in 2021 (Gütschow et al. 2021). Despite higher historical emissions, we expect lower growth rate in the coming decade, which could lead to peaking emissions before 2030. This is attributable to the adoption of additional policies, especially those aimed to reduce coal use (G. Liu et al. 2021; Tong et al. 2018)
EU27 + UK	− 20%	− 5%	The rate of emission decline has accelerated in the EU27 + UK, but COVID-19 is also a contributor of lower 2030 emissions. Updated emission projections are attributable to the adoption of new policies, which are reflected in updated data sources used as input for projections (EEA 2020; European Commission 2021). The effect of these policies can also be observed in emissions pre-2020
India	− 30%	− 10%	Emissions are still expected to increase but at a slower pace than initially projected. This is also observed in historical emissions. This change can be attributed to lower energy demand projections and faster renewable electricity uptake, displacing some of the country’s coal-fired electricity, which remain high (Dubash et al. 2018; Jones 2021). COVID-19 is also a major contributor of observed changes
Indonesia	5%	− 15%	Historical emissions increased substantially since 2015. The latest available year in official GHG inventory back in 2015 was 2000. The use of governmental projections as historical data resulted in underestimation of actual historical emissions (Government of Indonesia 2011). Projections now are in part determined by the 10-year electricity supply plans released by the state-owned electricity utility. The latest plan, which now covers the whole period until 2030, indicates a continued dependency on coal (Republic of Indonesia 2021)
Japan	− 5%	− 5%	Japan’s updated projections are only slightly lower than previously estimated. Our estimates are now lower mainly due to higher renewable shares in Japan’s electricity mix and the effect of the COVID-19 pandemic. The range is also narrower due to less uncertainty about nuclear future development
South Korea	− 10%	− 10%	The expected emission growth for the coming decade has not changed significantly, but the COVID-19 pandemic resulted in substantial emission reductions. Future emissions are highly dependent on the implementation of the Korean Emissions Trading Scheme, which could set emissions in a clearer downwards trajectory
Mexico	− 10%	− 10%	Updated historical data and the COVID-19 pandemic explain the lower emissions observed in 2030. However, emissions are expected to grow faster in the coming decade. Mexico modified its Electrical Industry Law in 2021 to allow certain fossil plants to obtain clean-energy certificates, which were previously planned for renewable energy suppliers (Diario Oficial de la Federación, 2021)
Russia	− 25%	− 5%	In Russia, future emission growth rates have been revised downwards, but emissions are still expected to increase. This reduction is not a result of additional climate policies. Russia has maintained their fossil-centred energy policy almost unaltered since 2015 (Mitrova & Melnikov 2019). A major revision to Russia’s emissions inventory explains most of the drop observed in 2030 emissions. This revision included an update to carbon dioxide and methane emission factors associated with fossil fuel exploration and production (Russian Federation 2019)
Saudi Arabia	− 45%	− 5%	Most recent projections show substantially lower emissions in 2030 in Saudi Arabia. Emissions have grown and are likely to grow much slower than expected since 2015. This is caused by COVID-19 and better estimates of the country’s projected energy demand (KAPSARC 2021). However, the country has made little progress on the implementation of its renewable energy targets (IRENA 2021)
South Africa	− 50%	0%	South Africa is the country with the most significant change in projections, which flipped from a substantial increase to a decrease in emissions up to 2030. This is driven by the planned decommissioning of most of the country’s coal fleet, as outlined in the latest Integrated Resource Plan published in 2019 (Department of Energy of the Republic of South Africa 2019). Reductions in economic growth expectations also contribute to the lower emissions in 2030
Turkey	− 35%	0%	Turkey’s expected emission change rate is lower today but remains the highest in the G20 group. The country continues to expand coal use in parallel to renewables, but many policies have been adopted since 2015 to support the letter (Jones 2021; Karapinar et al. 2019). The reduction observed is largely driven by changes in macro-economic forecasts, which now assume lower economic growth (IMF 2021; Republic of Turkey Ministry of Environment and Urbanization 2019)
United States	− 10%	0%	Emissions are expected to grow slightly slower than projected in 2015. Projections in the country have not been consistently revised downwards due to policy rollbacks introduced by the Trump administration (Jotzo et al. 2018). Despite the improvements observed since President Biden took office, current policies are insufficient to put emissions on a clear downwards trajectory

In 2020, COVID-19 resulted in drastic emission reductions worldwide. This drop is induced by restrictions in emission-intensive activities, such as aviation, urban mobility, and industrial production. Other researchers have analysed in detail the short-term impact of these restrictive measures on emissions across countries (Le Quéré et al. 2020, 2021; Z. Liu et al. 2020). The drop in emissions between 2019 and 2020 is significantly different from the national trend in almost all G20 countries (Tab S2). The pandemic also affects projections up to 2030 due to updated macro-economic forecasts and policy responses. We find that emissions are collectively 1.4 GtCO_2_eq (range: 1.3–1.6 GtCO_2_eq) lower in 2030 due to COVID-19. This corresponds to a 3.6% (range: 3.1–4.2%) reduction compared to emission projections excluding the effect of COVID-19. Our results are within that range of other studies, which find that COVID-19 policy responses and economic slowdown could reduce annual global emissions by 1–5 GtCO_2_eq, or 1.5–8.5%, in 2030 (Lecocq et al. 2022). The reduction in 2030 emissions explained by COVID-19 is almost one-quarter of the total reduction observed when comparing 2021 projections to 2015 projections.

However, the level of the reductions in 2030 emissions explained by COVID-19 varies across countries (‘2030 change COVID-19’ in Table 1). In most countries, COVID-19 explains less than one-third of the reductions in 2030 emissions between projections prepared in 2015 and 2021 (‘2030 change’ in Table 1). In some countries, COVID-19 explains reductions in the same order of magnitude of the overall reductions observed in 2030 emissions. This is the case for Japan, Mexico, and South Korea. In India, about one-third of the 30% reduction is explained by the pandemic, and in Indonesia, the only country with an increase in 2030 emissions between 2015 and 2021, emissions would be substantially higher if not for the economic slowdown induced by COVID-19.

Updates in historical data also influence emissions up to 2030 because projections are harmonised to the latest available historical year. The availability of additional and improved data sources increases the robustness of estimates. GHG emission inventory methodology improvements may result in shifts in the historical data series across the whole period analysed. More up-to-date historical data also include recent developments, such as the effect of adopted policies or short-term changes in the emission drivers. The attribution of the resulting changes to specific factors is challenging since there are many overlapping effects. However, they essentially result from the inclusion of recent developments. Back in 2015, historical data was scarce and outdated for some countries; the latest historical data points were on average 4 years old (median: 2012, range: 2000–2013). In 2021, data was on average 2 years old (median: 2019, range: 2016–2020). In most G20 countries, pre-2020 emissions are substantially different between the two sets of projections.

Post-2020 emission projections are influenced by the adoption of additional policies, especially those that reduce energy-related emissions. Since 2015, energy-related emissions have been periodically analysed by international organisations, such as the International Energy Agency (IEA). The IEA often revises their estimates to account for lower energy demand and higher rate of uptake of renewables (Fazendeiro & Simões 2021). These revisions partly result from additional energy efficiency and renewable energy policies (Table 1). Even though research indicates that forecasts might still underestimate renewable energy developments (Carrington & Stephenson 2018), latest projections result in lower 2030 projections in many G20 countries. Global developments to limit the use of coal also influence emission projections (Jewell et al. 2019; Rauner et al. 2020). This is relevant in both Australia and South Africa, countries with decisive changes in emission projections.

Information availability also affects our emission projections. Some G20 countries (Australia, Canada, and the EU27 + UK) publish official projections that consistently update the list of policies included as well as their effect on future emissions. New or additional scenarios became available for all countries analysed since 2015. This results from more transparent and frequent communication of climate change mitigation progress by non-Annex I countries that now submit Biennial Update Reports (BURs) to the UNFCCC. In ten G20 countries, most recent projections include additional sources.

## Discussion

The periodic evaluation of progress is a fundamental element of the Paris Agreement ambition-raising mechanism. The official Global Stocktake focuses on global emission projections and collective progress (UNFCCC 2022), but ultimately, national governments need to continuously update their NDCs as well as policies and actions to support them.

The G20 covers a large share of global emissions, and their emission projections bear a strong influence on global progress. We find that emissions of the G20 as a group are projected to increase up to 0.6% per year between 2021 and 2030. An increase in the emissions of group covering such as high share of global emissions is certainly misaligned with the goals of the Paris Agreement (IPCC 2018). Global emissions should fall by more than 7% per year between 2020 and 2030 (Höhne et al. 2020; UNEP 2019). Our analysis unpacks global trends and informs countries in updating their own policies and actions. We find that 2030 emissions are lower in most G20 countries when we compare 2021 to 2015 projections and that countries have often accelerated their efforts since 2015. Most countries with decreasing emissions are expected to decrease them faster and countries with increasing emissions, to increase them slower. National emissions change in the right direction.

Since the CAT analysis builds on or reviews many studies, the comparison to other literature is not trivial. Other country-specific analyses used as input to the CAT analysis show progress in the same direction and of similar magnitude to ours. Latest official projections for Australia and Canada show that emissions in both countries are approximately 40% and 30% lower compared to the 2015 projections, respectively (Australian Government 2015, 2021; Government of Canada 2021). Third-party estimates for the United States (Larsen et al. 2016; Pitt et al. 2021) show 2021 emission projections 10% below (calculation based on middle of the range) those projected in 2016. Energy-related CO_2_ emissions published by the IEA in their World Energy Outlook reports are also reviewed and often included in the CAT analysis. They show emissions lower than projected in 2015, except for Russia and China. Our 2021 estimates for Russia are lower than 2015 due to a revision in historical methane emissions, which are outside IEA’s scope. The IEA show an overall increase in energy-related CO_2_ emissions for approximately 10% for the same period analysed in this study. Our results for China indicate emissions have not changed substantially, but the IEA indicates an increase of approximately 5% in energy-related CO_2_ emissions. The IEA scenario is used as part of the upper range of our analysis but is adjusted downwards to better reflect key national energy policies, such as the target to meet 20% non-fossil energy share in 2025 (which was missed by 3% in the IEA analysis). For all other countries analysed, IEA emission projections are also expected to be lower than projected in 2015.

Even though national estimates for all G20 countries are unavailable, the UNEP Emissions Gap Reports and the Global Energy and Climate Outlook annually publish adopted policy emission projections (Keramidas et al. 2021; Labat et al. 2015; UNEP 2015, 2021). We compared 2015 and 2021 analyses from both groups and found that their 2030 emission projections are now also lower than projected in 2015, even though they estimate a lower effect.

This is in part due to the method used by these reports. The UNEP Emissions Gap Report includes peer-reviewed literature, which is often a few years behind in terms of policy cut-off date and in some cases did not include the effect of the COVID-19 pandemic. Höhne et al (2020), which builds on the work prepared for the UNEP Emissions Gap Reports, compare projections under current policies for seven major emitters to find that 2030 emissions were lower in 2019 than projected in 2015 for the EU27 and India, at a similar level in China, the United States, and Russia, and higher in Indonesia and Brazil. The differences between the findings of Höhne et al (2020) and our work are attributed to the inclusion of most recent trends. For example, our analysis includes the revision in inventory and COVID-19 impact for Russia and Biden’s administration reinstatement of some climate and energy for the United States. In the case of Brazil, the exclusion of LULUCF emissions in our projections influences the results, since LULUCF emissions have increased. Similar differences exist between our analysis and the 2021 version of Global Energy and Climate Outlook Report, which has 2019 as a policy cut-off date. Even though the report includes the effect of COVID-19, they do not reflect latest policies adopted. Nonetheless, we compared the G20 emission projections to 2030 and find that they are at least 3.5 GtCO_2_eq lower comparing the 2015 and 2021 versions of the report. Argentina, Australia, and Saudi Arabia were excluded from the 2015 version of the Global Energy and Climate Outlook Report. Including these countries would probably result in even higher emissions reductions.

Although our approach demonstrates clear trends in the G20 countries, it is subject to distinct limitations. Information availability, which substantially affect emissions projections, varies across countries. The implementation of common reporting tables and timeframes for all countries to submit data to the UNFCCC will improve data reporting and support the development of more consistent historical emission datasets and subsequently more accurate emission projections under current policies (Mayer 2019; Rajamani & Bodansky 2019; Streck et al. 2019). Changes in data availability will likely become less frequent over time, considering the significant improvements achieved in the past years. The convergence of emission inventory methodologies and reporting years reduces uncertainty associated with historical data updates. As new historical data becomes available, the effect of COVID-19 in the short-term also becomes less uncertain (Friedlingstein et al. 2021; IEA 2021a, 2021b; Z. Liu et al. 2022). However, COVID-19 policy responses and economic implications are likely to remain relevant for the next several years.

Even when these improvements are considered, the attribution of the changes in emission projections to the adoption of climate policies remains challenging. The methods used in our research do not allow to comprehensively unpack the effects of distinct factors explaining changes in emission trends. Studies that analyse the effect of policies on emissions often rely on statistical learning methods applied to many countries to establish associations between policies and emission trends (e.g. Eskander & Fankhauser 2020; Lachapelle & Paterson 2013; Le Quéré et al. 2019). They provide empirical evidence of policy effect but cannot clearly single out the effect of specific policies or factors. Studies that investigate the effect of policies on specific mitigation options complement these analyses and help to translate high-level findings into policy advice (Carley 2009; Green 2021). Others discuss the role of institutions, actors, and national process in enabling policies’ effect (Aklin & Urpelainen 2013; Lamb & Minx 2020). We argue that none of these can alone unpack climate policies’ effect but that their combination is conducive to understanding the effect of adopted policies and improving global mitigation efforts.

Despite these limitations, the estimates here represent our best understanding of the situation at the time, given the difficulties with methods and different sets of adopted policies. Our best estimate is now lower emissions in 2030 than we projected in 2015. This constitutes an improvement, regardless of the exact individual effects of the different causes of change.

## Conclusions

Policies adopted today affect GHG emissions for years and influence future climate-change levels. The urgency of the challenge to limit the global temperature increase to 1.5 °C demands a faster and stronger international response. To analyse projected and not only historical emissions helps to identify early issues with current mitigation efforts and steer towards meeting the international collective goals. Our research compares emission projections under adopted policies developed in 2015 to those prepared in 2021 to assess progress since the adoption of the Paris Agreement.

Our best available estimate of emission projections for the G20 has improved since 2015 and is now approximately 6 GtCO_2_eq lower in 2030. The most substantial changes are observed in India, the EU27 + UK, the United States, Russia, Saudi Arabia, and South Africa. In all these countries, COVID-19 results in a substantial (higher than 5%) drop in estimated emissions between 2019 and 2020. Pandemic-induced policy responses and economic slowdown also reduce projected emissions in almost all countries and explains approximately one-quarter of the reduction in 2030 emissions observed in the G20. In most recent projections, Australia, Canada, the EU27 + UK, Japan, South Korea, South Africa, and United States are expected to reduce their emissions below 2015 levels by 2030. Even though revised representation of historical effects and the pandemic influence our estimates, the adoption of additional policies explains lower emissions. Renewable uptake has been faster than projected before, efficiency improvements result in lower energy demand, and coal phase-out policies result in substantial shifts in emission projections of some countries. These factors combined result in changes in most countries’ 2021 projections when compared to those developed in 2015:

- South Africa presents the most significant change in emission projections in the coming decade. The country has improved from substantial annual increase on its emissions to an annual decrease between 2021 and 2030.
- The EU27 + UK and Japan were already expected to decrease their emissions in the coming decade but are expected to decrease it faster in 2021 projections. South Korea is likely to slightly decrease its emissions in the coming decade.
- The United States, Canada, and Australia were expected to increase their emissions and now show a slightly declining trend. This is a positive development, but their emissions per capita remain among the highest in the G20.
- China’s 2030 emissions remain at similar levels than projected in 2015. The faster historical emission growth is offset by slower emission growth in the coming decade. Emissions are now expected to peak before 2030.
- Argentina, Brazil, Mexico, Russia, and Saudi Arabia now show lower projected emissions in 2030 than estimated in 2015 but are expected to still increase their emissions. The implementation of Saudi Arabia’s renewable targets could result in plateauing emissions before 2030.
- Turkey slowed their substantial expected emission growth, but emissions increase up to 2030. Emissions in India and Indonesia also increase but remain at low per capita levels (below 5 tCO_2_eq) in 2030.

Emission projections in the G20 shows signs of improvement, but progress remains slow compared to what is needed under the Paris Agreement. Emissions of the G20 as a group are expected to slightly increase in the coming decade. No single G20 country shows rates of emissions decline in line with the necessary global rate to meet the goals of the Paris Agreement. Projected annual growth rates also do not differ statistically when compared to the previous decade. We do not observe the transformative change necessary to reach the global temperature goals. The G20 remain off track to curb their emissions before 2030.

Our research shows that progress is slow in major emitting countries but also that pivotal shifts in emission trajectories took place since the adoption of the Paris Agreement. Countries with increasing emissions were able in half a decade to substantially reduce their projected growth or even put emission projections in a downwards trajectory. Scenarios presented here estimate the effect of adopted policies and do not constitute a fixed, definite trajectory for the coming decade. Decisions that shape future climate are, and need to be, made today. The G20 group must urgently and drastically improve adopted policies and actions to limit the end-of-century warming to 1.5 °C.

## Supplementary Information

Below is the link to the electronic supplementary material.

Supplementary file1 (DOCX 488 KB)

## Data Availability

The data used for the current policy scenario is publicly available for non-commercial purposes at www.climateactiontracker.org. All other data supporting our findings are included in the manuscript.

## Footnotes

1 We consider the European Union member states as one under the EU27 + UK. In this research, the UK is considered part of the EU due to its membership status in 2015. We use the term country to refer to the ‘EU27 + UK’ region.
