# Supplementary material for: The G20 emission projections to 2030 improved since the Paris Agreement, but only slightly
Source: Mitig Adapt Strateg Glob Chang. 2022 Jul 14;27(6):39. doi: 10.1007/s11027-022-10018-5 (PMC9281192; doi:10.1007/s11027-022-10018-5)
Supplement: Supplementary file 1 — Supplementary file1 (DOCX 488 KB) [file 11027_2022_10018_MOESM1_ESM.docx]

Supplementary material: The G20 emission projections to 2030 improved since the Paris Agreement, but only slightly

Historical data

The latest historical year varies across countries due to distinct reporting requirements (Tab 1). Historical emissions data was primarily based on reports by national governments submitted to the UNFCCC, such as national communications (NCs), national inventories reported in common table format (CRF) for Annex I Parties and Biennial Update Reports (BURs) for non-Annex I Parties. These reports were supplemented by other estimates to provide up to date and complete historical time series (Gütschow et al., 2016).

Tab S1: Data sources for historical data used in 2015 and 2021 projections.

| Country | 2015 | | 2021 | |
| --- | --- | --- | --- | --- |
|  | *Last reported year* | *References* | *Last reported year* | *References* |
| Argentina | 2012 | NC3 | 2016 | BUR3 |
| Australia | 2012 | CRF 2014 | 2020 | (DISER, 2021) |
| Brazil | 2012 | GHG Inventory +  SEEG 2014 | 2020 | BUR3 +  SEEG 2021 |
| Canada | 2012 | CRF 2014 | 2019 | CRF 2021 |
| China | 2010 | (CDIAC, 2012; IEA, 2014; US EPA, 2012) +  GHG Inventory | 2019 | PRIMAP + GCP |
| EU27 + UK | 2012 | CRF 2014 | 2019 | (EEA, 2021) |
| India | 2010 | (CDIAC, 2012; IEA, 2015; US EPA, 2012) +  GHG Inventory | 2019 | PRIMAP + GCP |
| Indonesia | 2000 | GHG Inventory | 2019 | PRIMAP + GCP |
| Japan | 2013 | CRF 2014 | 2019 | CRF 2021 |
| Mexico | 2010 | NC5 | 2017 | (INECC, 2018) |
| Russia | 2012 | CRF 2014 | 2019 | CRF 2021 |
| Saudi Arabia | 2012 | GHG Inventory | 2019 | PRIMAP + GCP |
| South Africa | 2010 | (DEA, 2013) | 2017 | (DEFF, 2021) |
| South Korea | 2012 | GHG Inventory | 2019 | PRIMAP + GCP |
| Turkey | 2012 | CRF 2014 | 2019 | CRF 2021 |
| United States | 2012 | CRF 2014 | 2019 | CRF 2021 |

Legend:

- **CRF**: Common Reporting Format: [National Inventory Submissions 2021 | UNFCCC](https://unfccc.int/ghg-inventories-annex-i-parties/2021)
- **BUR #**: Biennial Update Report for non-Annex-I countries: [Biennial Update Report submissions from Non-Annex I Parties | UNFCCC](https://unfccc.int/BURs)
- **GHG Inventory**: UNFCCC Greenhouse Gas Emissions Inventory: [Greenhouse Gas Inventory Data - Time Series - Annex I (unfccc.int)](https://di.unfccc.int/time_series)
- **PRIMAP**: The PRIMAP-hist national historical emissions time series: [Paris Reality Check: PRIMAP-hist (pik-potsdam.de)](http://www.pik-potsdam.de/paris-reality-check/primap-hist/#scenario=histcr&id=earth&entity=kyotoghgar4)
- **NC #**: National Communications for non-Annex-I countries: [National Communication submissions from Non-Annex I Parties | UNFCCC](https://unfccc.int/non-annex-I-NCs)
- **GCP:** Global Carbon Budget project: [GCP - Carbon Budget (globalcarbonproject.org)](https://www.globalcarbonproject.org/carbonbudget/21/data.htm)
- **SEEG:** Sistema de Estimativa de Emissões de Gases de Efeito Estufa: [Total Emissions | SEEG - System Gas Emissions Estimation](https://plataforma.seeg.eco.br/total_emission)

Other supplementary figures and tables


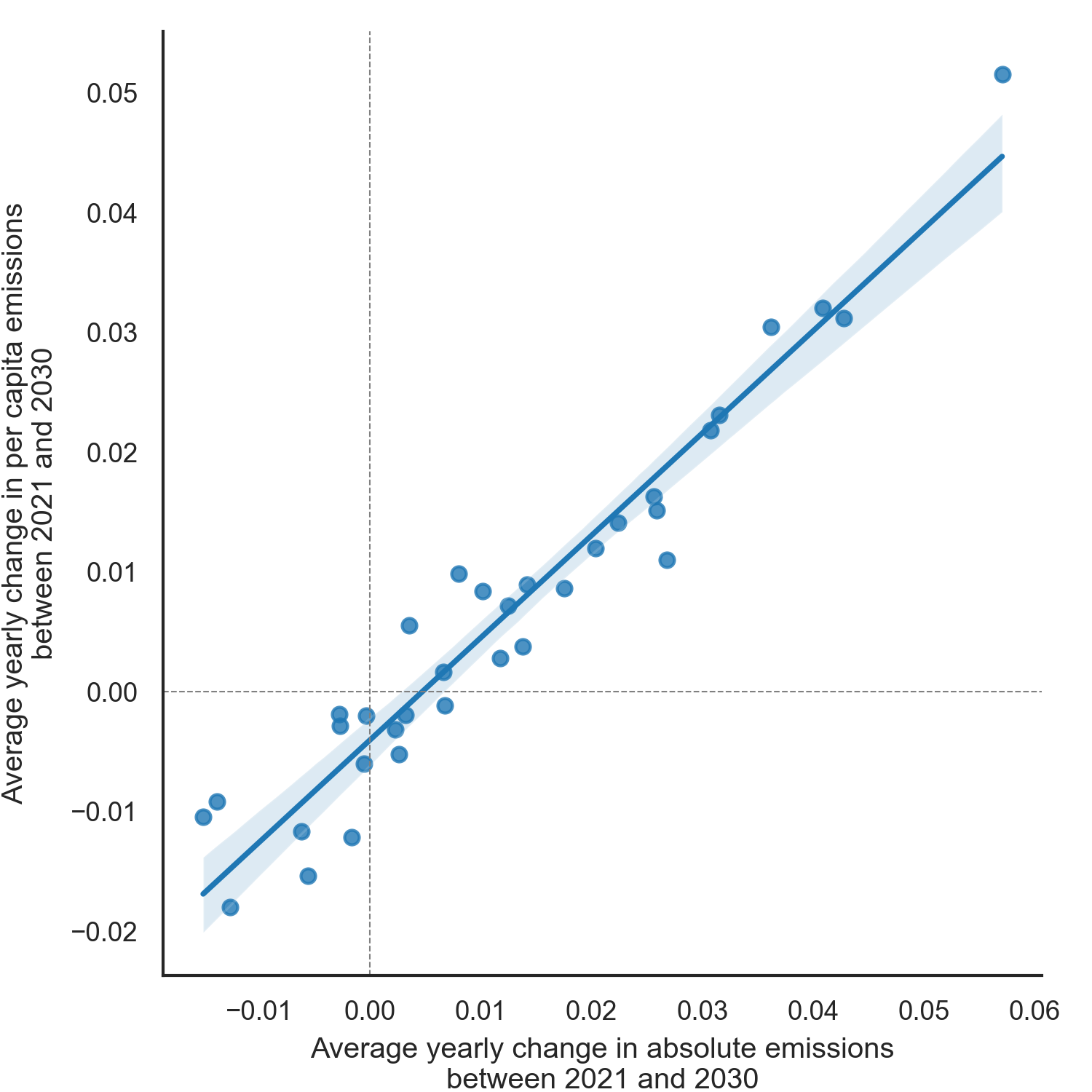


Fig S1: Comparison of emissions rate per capita and absolute. Each dot represents one of the G20 countries. Robust relationship (p-value < 0.001).


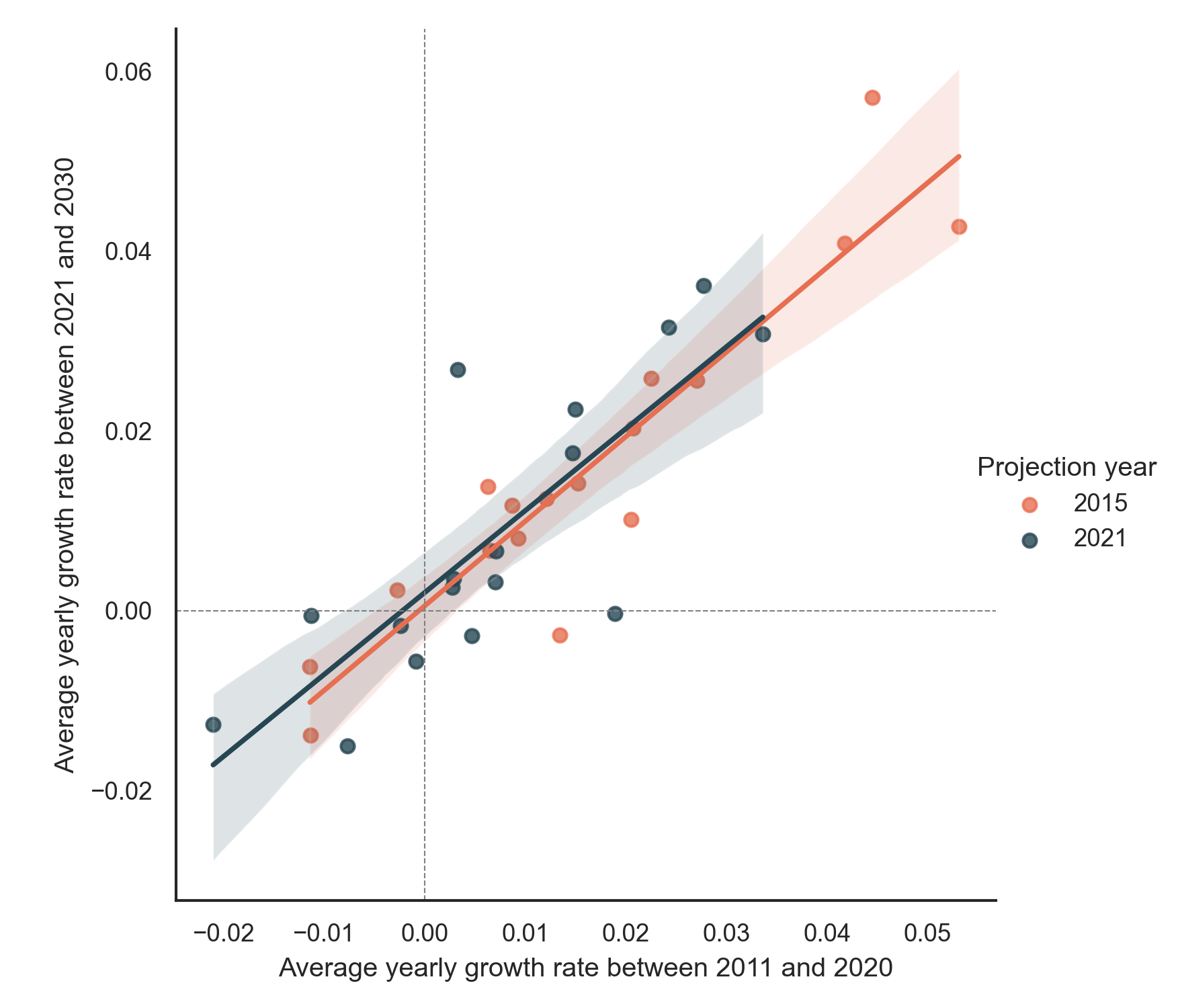


Fig S2: Comparison of average growth rates per country in the 2010s and 2020s. Each dot represents one of the G20 countries. The two distributions are not statistically different (p-value > 0.1).

Tab S2: Countries with increase or decrease (above 3%) in historical emissions trajectories per period analysed. We use ‘significant’ for differences larger than 5%. For changes ‘before 2010’ and ‘between 2010 and 2019’, the percentage differences are calculated by comparing 2021 to 2015 projections. To estimate the effect of COVID-19 historical drop we calculate the difference between the annual change rate in 2020 to the average annual change rate in the decade before.

| Country | Changes before 2010 | Changes between 2010 and 2019 | COVID-19 effect in 2020 |
| --- | --- | --- | --- |
| EU27+UK | - | Significant decrease | Significant decrease |
| ARG | Significant decrease | Significant decrease | Significant decrease |
| AUS | Decrease | Decrease | - |
| BRA | - | Decrease | Decrease |
| CAN | - | - | Significant decrease |
| CHN | Significant increase | Increase | - |
| IND | Significant decrease | Decrease | Significant decrease |
| IDN | Increase | Significant increase | Decrease |
| JPN | Increase | Decrease | Decrease |
| KOR | - | Decrease | Significant decrease |
| MEX | Significant decrease | Significant increase | Significant decrease |
| RUS | Significant decrease | - | Significant decrease |
| SAU | Increase | Significant decrease | Decrease |
| ZAF | Significant decrease | Significant decrease | Significant decrease |
| TUR | - | Significant decrease | - |
| USA | - | Decrease | Significant decrease |
| Significant in # countries | 6 | 7 | 9 |

References

CDIAC. (2012). *Global, Regional, and National Fossil Fuel CO2 Emissions*. http://cdiac.ornl.gov/trends/emis/meth_reg.html

Christoff, P., & Eckersley, R. (2021). Convergent evolution: framework climate legislation in Australia. *Climate Policy*, *21*(9), 1190–1204. https://doi.org/10.1080/14693062.2021.1979927

Crowley, K. (2021). Fighting the future: The politics of climate policy failure in Australia (2015–2020). *WIREs Climate Change*, *12*(5), e725. https://doi.org/https://doi.org/10.1002/wcc.725

DEA. (2013). *GHG Inventory for South Africa* (Issue August). https://www.environment.gov.za/sites/default/files/docs/greenhousegas_invetorysouthafrica.pdf

DEFF. (2021). *National GHG inventory report South Africa 2000 - 2017*. https://www.environment.gov.za/sites/default/files/docs/nir-2017-report.pdf

Department of Energy of the Republic of South Africa. (2019). *Integrated Resource Plan (IRP2019)*. https://www.gov.za/sites/default/files/gcis_document/201910/42778gon1359.pdf

Diario Oficial de la Federación. (2021, March 9). *Decreto por el que se reforman y adicionan diversas disposiciones de la Ley de la Industria Eléctrica [Decree amending and adding various provisions of the Electricity Industry Act]*. https://www.diputados.gob.mx/LeyesBiblio/pdf/LIElec_090321.pdf

DISER. (2021). *Quarterly Update of Australia’s National Greenhouse Gas Inventory: September 2020*. https://www.industry.gov.au/sites/default/files/2021-02/nggi-quarterly-update-september-2020.pdf

EEA. (2021). *EEA greenhouse gas - data viewer*. https://www.eea.europa.eu/data-and-maps/data/data-viewers/greenhouse-gases-viewer/

Gütschow, J., Günther, A., & Pflüger, M. (2021). *The PRIMAP-hist national historical emissions time series v2.3 (1750-2019)*. https://doi.org/10.5281/zenodo.5175154

IEA. (2014). *World Energy Outlook*. https://www.iea.org/publications/freepublications/publication/WEO2014.pdf

IEA. (2015). *World Energy Outlook 2015*. Paris, France: International Energy Agency.

INECC. (2018). Inventario Nacional de Emisiones de Gases y Compuestos de Efecto Invernadero 1990-2015 en México. *Instituto Nacional de Ecología y Cambio Climático*, 845.

Jones, D., Graham, E., Tunbridge, P., & Ilas, A. (2020). *Global Electricity Review 2020* (Issue March). EMBER.

Nascimento, L., Kuramochi, T., Iacobuta, G., den Elzen, M., Fekete, H., Weishaupt, M., van Soest, H., Roelfsema, M., De Vivero-Serrano, G., Lui, S., Hans, F., Jose de Villafranca, M., & Höhne, N. (2021). Twenty years of climate policy: G20 coverage and gaps. *Climate Policy*. https://doi.org/10.1080/14693062.2021.1993776

Republic of Indonesia. (2021). *Rencana Usaha Penyediaan Tenaga Listrik (RUPTL) 2021-2030*. https://web.pln.co.id/statics/uploads/2021/10/ruptl-2021-2030.pdf

Republic of Turkey Ministry of Environment and Urbanization. (2019). *Turkey’s Fourth Biennial Report*. https://www4.unfccc.int/sites/SubmissionsStaging/NationalReports/Documents/9645137_Turkey-BR4-1-FOURTH BIENNIAL REPORT OF TURKEY.pdf

Ruggeri, E., & Garrido, S. (2021). More renewable power, same old problems? Scope and limitations of renewable energy programs in Argentina. *Energy Research & Social Science*, *79*, 102161. https://doi.org/https://doi.org/10.1016/j.erss.2021.102161

Russian Federation. (2019). *Russia’s 4th Biennial Report*. https://unfccc.int/sites/default/files/resource/124785_Russian Federation-BR4-2-4BR_RUS_rev.pdf

US EPA. (2012). *Global Anthropogenic Non-CO2 Greenhouse Gas Emissions: 1990-2030. Revised December 2012*. Office of Atmospheric Programs Climate Change Division, U.S. Environmental Protection Agency. http://www.epa.gov/climatechange/Downloads/EPAactivities/EPA_Global_NonCO2_Projections_Dec2012.pdf
